# Growth Hormone and Reproduction: A Review of Endocrine and Autocrine/Paracrine Interactions

**DOI:** 10.1155/2014/234014

**Published:** 2014-12-15

**Authors:** Kerry L. Hull, Steve Harvey

**Affiliations:** ^1^Department of Biology, Bishop's University, Sherbrooke, QC, Canada J1M 1Z7; ^2^Centre de Recherche Clinique Etienne-Le Bel, Université de Sherbrooke, Sherbrooke, QC, Canada J1H 5N4; ^3^Department of Physiology, University of Alberta, Edmonton, AB, Canada T6G 2R3

## Abstract

The somatotropic axis, consisting of growth hormone (GH), hepatic insulin-like growth factor I (IGF-I), and assorted releasing factors, regulates growth and body composition. Axiomatically, since optimal body composition enhances reproductive function, general somatic actions of GH modulate reproductive function. A growing body of evidence supports the hypothesis that GH also modulates reproduction directly, exerting both gonadotropin-dependent and gonadotropin-independent actions in both males and females. Moreover, recent studies indicate GH produced within reproductive tissues differs from pituitary GH in terms of secretion and action. Accordingly, GH is increasingly used as a fertility adjunct in males and females, both humans and nonhumans. This review reconsiders reproductive actions of GH in vertebrates in respect to these new conceptual developments.

## 1. Introduction

We previously published a series of comprehensive reviews of GH and reproduction in 2000–2002 [1–4]. Like these earlier works, the present monograph integrates data from clinical, agricultural, and experimental studies. In addition to incorporating recent articles, we have reinterpreted the role of GH in reproduction in light of two major conceptual developments: firstly, that autocrine/intracrine GH may exert distinct roles from endocrine GH and, secondly, that GH may have detrimental effects on neoplasm development and insulin resistance. We do not discuss mammary gland GH, since its production and action have been comprehensively and periodically reviewed in the past decade [5–10].

## 2. An Updated View of the Mechanism of Action of GH

The classical somatomedin view of GH action, in which GH of pituitary origin acts at membrane receptors to stimulate hepatic IGF-I production, which, in turn, alters organ growth, has been significantly modified in the last 20 years. These changes have significant implications for understanding reproductive GH actions, so they will be briefly reviewed here.

Firstly, the revised hypothesis retains the GH-dependence of hepatic IGF-I, but this endocrine IGF-I is strictly required only for the feedback regulation of GH secretion [11, 12]. While GH-induced hepatic IGF-I production is still relevant to its somatic effects, GH-induced IGF-I production within GH-target tissues may be equally or more important. To further complicate the picture, IGF-I production in newly discovered GH target sites such as the brain, heart, and reproductive organs is largely GH-independent and is instead controlled by other factors such as gonadotropins or estradiol [13]. Thus, older studies indicating that reproductive GH actions are mediated by hepatic IGF-I need to be revisited.

Secondly, GH can activate or induce other receptors with proven neoplastic effects. GH can activate heterodimers consisting of the GHR and the prolactin receptor (PRLR) in breast tissue, activating PRL signaling pathways [14], and GHR-IGF-1R heterodimers may potentiate GH signaling in prostate cancer cells [15]. GH also induces EGF receptor expression [16] and GH can also indirectly activate the EGF receptor (EGFR) and activate signaling pathways in preadipocytes [17].

A third major paradigm shift is the distinct secretory patterns and actions of locally produced and circulating GH. While the pituitary gland remains the primary source of circulating GH, GH is also produced within reproductive cells (reviewed by [18]). Unlike the sexually dimorphic pulsatile nature of pituitary GH secretion [19], extra pituitary GH is produced more continuously and at lower levels [10]. Newly synthesized GH can bind GHRs in the endoplasmic reticulum, and the resulting GH:GHR complexes travel to the cell surface and activate the JAK-STAT pathway [20]. The continuous activation induced by local GH promotes a different pattern of gene expression and cell growth than systemic GH [21]. As discussed later, this distinction may underlie the increased tumorigenic potential of local GH compared with endocrine GH [22].

## 3. Detrimental GH Actions

The reduced incidence of cancer in humans with Laron Syndrome [23] and in GHR knockout mice [24] suggests that GH may exert neoplastic effects. However, these results likely reflect resistance to the autocrine, rather than endocrine, actions of GH. Elevated systemic GH does not appear to be oncogenic, since the overall cancer incidence is normal in acromegalics [25] and is not increased by GH treatment of GHD children and adults [26, 27]. Extrapituitary GH, conversely, may act as a “one-step oncogene” [9] within the producing cells, promoting both proliferative and metastatic processes in sites such as the breast and prostate gland [28–30]. The relevance of autocrine GH to neoplasms has been extensively reviewed in relevance to mammary GH [9] and will be discussed in the context of prostate GH below. Thus, GH administration in clinical and agricultural settings does not appear to increase the cancer risk.

Interactions between GH and insulin are also relevant to any consideration of therapeutic GH uses. Chronic GH overexposure may increase the incidence and severity of diabetes mellitus, since this chronic disorder is more prevalent is acromegalics and improves with treatment of the GH excess [31–33]. While the data is somewhat obscure, it appears that GH also contributes to insulin resistance and impacts glucose control in type I diabetics [33].

## 4. Hypothalamic-Pituitary Interactions

It is increasingly evident that GH modifies numerous aspects of hypothalamic function via hypothalamic GH receptors [34]. Neuroendocrine interactions have, for instance, been implicated in the reduced responsiveness to pheromonal stimuli in GHR-KO mice [35]. However, in relation to reproduction it appears unlikely that GH modulates hypothalamic GnRH release; instead, GH acts at pituitary and gonadal sites to modify GnRH actions.

Pituitary somatotrophs and gonadotrophs are, in part, both coregulated and interdependent. Kisspeptin, a potent GnRH-releasing factor, stimulates both LH and GH release from peripubertal rat pituitary cells [36]. PACAP similarly regulates both cell types [37]. Moreover, a subset of rat pituitary cells secrete both gonadotropins and GH [38], and some studies show that gonadotroph development is GH-dependent [4].

Some reproductive actions of GH are, therefore, likely mediated at the pituitary level. Gonadotrophs contain GH receptors (GHRs) and/or GH binding proteins (GHBPs) and LH/FSH secretion is reduced in GH-deficient/resistant rats [4]. The effects of exogenous GH are not, however, as clear-cut. Depending on the species and reproductive state of the animal, GH exerts stimulatory, inhibitory, or minimal effects on LH and/or FSH secretion [4]. Sirotkin [39] suggests that gonadotropin secretion in rodents is more sensitive to GH modulation than that in ruminants and primates.

## 5. Puberty

The dramatic transformations of puberty include sexual maturation and accelerated growth. Complex interactions between the somatotropic and gonadotrophic axes govern these two interrelated processes. Puberty begins with the activation of the GnRH pulse generator, but the factors activating the pulse generator are complex and species-specific. The somatometer hypothesis implicates nutritional and growth signals in the timing and tempo of puberty [40], and GH, IGF-I, and leptin may be the signals for adequate nutrition (reviewed by [41]).

Since GHR-KO mice (unlike IGF-I-KO mice) do sexually mature and are somewhat fertile, GH is not absolutely required for pubertal development [42]. Instead, data suggests that GH modulates the timing of sexual maturation (reviewed by [43]). For instance, the timing of both the vaginal opening and the first pregnancy is delayed in female GHR-KO mice [42, 44], and expression of the GH transgene hastens the pubertal onset [45]. The onset of puberty is similarly delayed in male GHR-KO mice, GH-deficient dwarf mice, and GH-deficient rats (reviewed by [43]). Human studies support these findings, since pubertal onset and/or menarche is delayed in GHD and GH-resistant children despite their increased adiposity [46, 47]. Conversely, GH suppression in monkeys prolongs puberty but does not alter the timing of pubertal initiation [48]. In children with idiopathic short stature, however, most studies do not reveal any impact of GH treatment on the age of pubertal onset or the pubertal duration ([49] and references therein). Curiously, some data suggest that the absence of GH action may delay the age-related decline in fertility [43].

GH may alter the timing of puberty by participating in the activation of the GnRH pulse generator [50]. This action is generally thought to be IGF-I mediated [3], but recent studies in GHD and GH-resistant children demonstrated that IGF-I could not normalize the timing of pubertal onset. However, in the studies of Sharara and Giudice [51], GH could only accelerate puberty when a pubertal pattern of pituitary gonadotropin secretion is established [51], and others implicate changes in FSH secretion and gonadal LH receptors [52] or gonadal steroidogenesis with altered GH action [53]. GH may also act at target sites, by potentiating androgen action [54].

The somatotropic-gonadotrophic interactions at puberty are bidirectional, since the dramatic pubertal changes in GH secretion are dependent upon sex steroids (particularly estrogens) [55, 56]. Normal pubertal growth depends on the resulting coordinated actions of growth hormone and the sex steroids (see, e.g., [57]).

## 6. Male Fertility

The male gonad and accessory organs are sites of both GH production and action. Since GHR-knockout mice have decreased (but not abolished) fertility [58], physiological GH levels are profertility, but GH may promote tumorigenesis, particularly in the prostate.

### 6.1. Testes

GH promotes testicular growth and development and stimulates gametogenesis and steroidogenesis in the adult testes.

#### 6.1.1. Growth and Development

GH promotes seminiferous tubule differentiation and supports normal testicular growth (reviewed by [2, 39]). IGF-I may mediate these actions, since it can rescue testicular differentiation in fetal mice treated with GH antibodies [59] and growth in GH-resistant boys [60]. While Lindgren et al. [61] observed normal testicular development in boys with GH deficiency or idiopathic short stature regardless of GH treatment status, a newer study observed increased testicular volume in GH-treated short children associated with a longer pubertal duration [49]. High-dose GH treatment of GH-replete animals conversely induces atrophy of the testes and accessory organs in dogs [62] and has no effect on testicular volume in monkeys [63]. Overexpression of the GH transgene similarly impairs testicular development (reviewed by [2]); thus, testicular actions of GH, like many other actions, appear to be biphasic.

#### 6.1.2. Steroidogenesis

GH is a potent steroidogenic factor, particularly* in vitro*. GH stimulates androgen and/or estradiol production by Leydig cells isolated from rodents, ruminants, humans, and fish [39, 64], but not horses [65]. The results of* in vivo* studies are more controversial. While chronic GH therapy improves chorionic gonadotropin- (CG-) induced testosterone production in some studies of fertile GH-deficient males [66, 67] and the testosterone response to hCG is attenuated in GHR knockout mice [52], experiments in monkeys [63], swine [68], and ruminants [68] failed to demonstrate an androgenic GH response. Indeed, GH treatment in hypopituitary or moderately obese men actually decreases the concentrations of total serum testosterone [69, 70], potentially due to a stimulatory effect on aromatase activity and the resulting conversion of testosterone to estradiol observed in healthy young men treated with GH [71].

*In vitro* studies also reveal that GH alters the activity of enzymes involved early in the steroidogenic pathway; it stimulates the production of steroidogenic acute regulatory protein (StAR), which mediates cholesterol translocation across the inner mitochondrial membrane, and 3-beta hydroxysteroid dehydrogenase, which converts pregnenolone into progesterone [53], in rat Leydig cell precursors. The formation of early steroidogenic intermediates, such as 17-alpha-20-beta dihydroprogesterone, is similarly upregulated by GH in fish testicular cells [72].

GH may potentiate gonadotrophic effects on steroidogenesis by enhancing testicular LH sensitivity and promoting Leydig cell development, since GHR knockout mice have fewer Leydig cells and LH receptors [52]. Similarly, GH upregulates LH receptors in both GH-replete (e.g., hamsters [73]) and GH-deficient (e.g., dwarf mice [74]) animals.

Sex hormone binding globulin (SHBG) reduces testosterone bioavailability. Some studies suggest that GH might potentiate testosterone action by decreasing SHBG production. For instance, GH therapy reduces SHBG concentrations in GHD adults in some [70, 75] but not all [76] studies and in hypopituitary boys [77]. The pubertal rise in GH production may potentiate male pubertal development, since the age-related decrease in SHBG concentration is not observed in GHD boys [78].

However, other studies in normal men reveal coordinated decreases in SHBG and total serum testosterone production following GH treatment [69], reduced SHBG but unchanged total serum testosterone [79], or increased LH-induced testosterone but unchanged SHBG [66]. These discrepancies may reflect differences in subject age and GH administration protocol.

Some investigators have implicated IGF-I in the steroidogenic actions of GH. IGF-I can mimic the effects of GH in rat testis [80] and partially restore testosterone synthesis in GH-resistant men [60]. Moreover, in the study of [81], GH-induced steroidogenesis required IGF-I coadministration. However GH-induced StAR synthesis does not require de novo protein synthesis, suggesting that at least some testicular actions are IGF-I independent [53].

The study of Ramdhan et al. [82] observed a correlation between testicular GHR expression and StAR and P450 expression following exposure to nanoparticle-rich diesel exhaust (NR-DE) in rats. However, more studies are required to identify a causal relationship between GH and pollutant-induced androgenesis.

#### 6.1.3. Gametogenesis

The impact of GH on testicular growth may reflect germ cell proliferation, particularly in situations that impair spermatogenesis. GH partially compensates for GnRH immunoneutralization by increasing the number of mature spermatids in prepubertal male rats [83]. In GH-deficient dwarf rats, GH prevents the decrease in spermatid count and spermatozoa motility resulting from treatment with cyclophosphamide, a chemotherapeutic drug with strong testicular toxicity [84]. Similarly, GH protects against the inhibitory effect of MTX on sperm count and motility, testosterone production, and testicular atrophy in GH-replete Wistar rats [85]. GH may thus be a useful adjuvant to chemotherapy regimens in order to preserve male fertility. However, GH overexpression in the testes of transgenic zebrafish reduces sperm motility, fertility, and the production of offspring [86], highlighting the importance of careful dosing.

GH also improves sperm morphology and motility in GH-deficient dw/dw rats [87] and prolongs overall equine spermatozoa motility* in vitro*, possibly by extending sperm longevity [88]. Moreover, GH gene polymorphisms are associated with numerous indicators of sperm quantity and quality in bulls [89]. Gametogenesis is similarly enhanced by GH in* in vitro *cultures of eel testicular cells [90]. In contrast, GH does not appear to enhance gametogenesis in many GH-replete animals, since GH supplementation does not alter germ cell apoptosis in bulls [91] nor sperm number in monkeys [63] and postpubertal rats [92]. Moreover, men with the GHR3d (3rd exon) deletion do not have improved semen quality or steroid production, despite the increased GH sensitivity conveyed by this mutation [93].

Many (but not all) azoospermic infertile men are relatively GH-deficient, as manifested by a reduced GH response to arginine and/or clonidine [94], and GH adjuvant therapy improves spermatogenesis and improves sperm motility in this subset of infertile men [95, 96]. Local application of GH appears to restore germ cell number and morphology [97]. However, other studies fail to show a beneficial effect of GH in gonadotropin-treated men (e.g., [98]), reflecting the highly heterogeneous etiology of male infertility.

Local IGF-I production may mediate spermatogenic effects of GH, since IGF-I can also improve sperm morphology and motility [87] and GH coordinately increases seminal IGF-I and sperm motility in some (but not all) studies (reviewed by [2]). However, some studies report discordant effects of GH and IGF-I [99], suggesting that GH may also act independently. Similarly, the stimulatory effect of GH on eel spermatogenesis is both IGF-I and steroid independent [90].

The reduced, but not abolished, fertility in GH-resistant men and mice and GH-deficient rats [42, 100, 101] suggests that enough GH-independent testicular IGF-I production occurs to enable a low degree of fertility. GH-independent testicular IGF-I production in chickens, conversely, appears to be at a sufficient level to completely restore fertility, since seminal IGF-I concentrations, sperm viability, motility, morphology, and fertility do not vary between GH-resistant and GH-replete chickens [102].

#### 6.1.4. The Testicular Minihypophysis

Circulating GH cannot readily access testicular cells within the blood-testis barrier, such as spermatids or spermatozoa. The ligands for the GHRs on these cells are thus likely to be produced within the testis. In support of this contention, GH gene expression has been detected within the rat, human, and chicken testis (reviewed by [2]) and, more recently, the eel testis [90]. Curiously, the GH-variant gene products, previously thought to be pregnancy-specific, are the most abundant GH mRNA isoform in the human testis [103]. Moreover, while GH-N gene products are detectable in both cancerous and normal testicular tissue, GH-V gene products are only detected in normal tissue [104].

The testis is the only detectable site of extrapituitary GH mRNA expression in pejerrey fish [105] and fathead minnows [106]. GH mRNA abundance increases with sexual development, at least in fathead minnows, highlighting the potential importance of local testicular GH production in piscines [106].

To our knowledge, GH-producing cell types have only been elucidated in chickens and eels. GH mRNA and immunoreactivity are largely absent from avian Sertoli and Leydig cells [107, 108]. Instead, GH mRNA is largely confined to spermatogonia and primary spermatocytes, but GH immunoreactivity is only detectable in secondary spermatocytes and spermatids [108]. Similarly, GH mRNA is abundant in germ cells in eel testes, but GH immunoreactivity is particularly abundant in the surrounding Sertoli cells [90].

Testicular GHR immunoreactivity and/or binding sites have been detected in male fetal and adult rats [109, 110]. GHR mRNA is similarly present in the testis of teleost fish [111], particularly in Sertoli cells [112], and in eels [90], particularly in developing gametes. In chickens, GHR gene expression has been detected in Sertoli, Leydig, and peritubular cells [113]. GHR immunoreactivity in the human testis, conversely, appears concentrated in Leydig cells [114]. Testicular GH-binding activity is not inconsequential, reaching 40% of hepatic levels in prepubertal boar testis [115].

The testicular GHR mRNA concentration decreases with sexual maturity in Nile Tilapia [116] and rainbow trout [112], in stark contrast to the increase observed in ovarian tissues [116]. The lower concentration of GHR mRNA in the testis than in the ovary of Tilapia implicates sex steroids in the regulation of testicular GH sensitivity [117]. However, other investigators have observed equal ovarian and testicular GHR mRNA concentrations in tilapia [118] or higher testicular levels in sea bream [119].

Potential GH regulators are similarly expressed in the testis. In rats and humans, for instance, testicular GHRH closely resembles placental GHRH and is capable of stimulating pituitary GH release and Sertoli cell adenylate cyclase activity (reviewed by [2]). GHRH receptors have a wide distribution in humans, including Leydig cells, Sertoli cells, germ cells, and the prostate gland, suggesting GHRH may exert testicular actions distinct from GH [120]. In chickens, GHRH is colocalized with GH in Leydig cells and tubular myocytes and more abundantly in germ-line cells [121]. GHRH receptors are also present in the chicken testes [122]. A recent study in chickens has shown that GHRH stimulates testicular GH secretion and alters posttranslational processing, increasing the abundance of short (15 kDa and 17 kDa) forms and decreasing the relative abundance of 21 kDa GH [121]. Exogenous GHRH stimulates testicular cell proliferation and PCNA production, an effect at least partially mediated by local GH since it is blocked by GH antibodies [121]. More recently, Ghrelin and putative Ghrelin receptors have been localized in the testis, and Ghrelin alters testosterone synthesis and other testicular parameters [123, 124].

The relevance of testicular IGF-I to gonadal function is well-established [125]; however, gonadotropins, rather than GH, may be its primary regulator [13, 43]. Testicular IGF-I in rats, for instance, responds poorly to changes in the systemic GH concentration [126]. GH stimulates IGF-I production in Leydig cells isolated from rat [81] but not horse [127]. In chickens, testicular IGF-I production appears to be entirely GH-independent, since it is elevated in GH-resistant dwarf chickens [128]. It may also be of interest to investigate the regulation of testicular IGF-II in teleosts, since it has been shown to be GH-dependent in other nonhepatic sites [129].

### 6.2. The Male Accessory Organs

The presence of GHRs in the male accessory organs [109, 110] suggests that reproductive actions of GH in the male are not confined to the testis. Indeed, Wolffian duct differentiation into the prostate gland and seminal vesicles in fetal rats is strongly influenced by GH [59].

Local GH production mediates this effect, since the pituitary GH synthesis is negligible at this early stage of embryonic development [130]. The postnatal activity of the prostate gland and other male accessory organs may be similarly dependent on GH, of pituitary or local origin.

#### 6.2.1. The Prostate

GH stimulates prostate growth, since the prostate and seminal vesicles are smaller in mice transgenic for a GH antagonist [131] and in Laron mice [132]. Similarly, GH stimulates prostate growth in GH-deficient rats [133] and prostatic enzyme production in immature GH-replete rats [35, 134]. Prostate hyperplasia and structural abnormalities (such as cysts, nodules, or calcifications) are more common in acromegalics [135]; thus, GH also stimulates prostate growth in humans. However, numerous epidemiological studies suggest that endocrine GH promotes prostate hyperplasia but is not neoplastic in this tissue. For instance, studies attempting to correlate endocrine GH status with prostate cancer risk have observed no relationship [136] or an inverse relationship [137]. PSA levels and the risk of prostate cancer are normal in acromegalics [138], and GH suppression in these patients decreases prostate volume but does not affect PSA levels [135]. Similarly, GH replacement in GHD adults increases prostate volume, particularly when coadministered with testosterone, without altering prostate-specific antigen expression or inducing morphological abnormalities [139, 140]. It has been hypothesized that endocrine GH induces hyperplasia (but not neoplasms) via hepatic IGF-I [29]. However, this hypothesis is counterindicated by the observation that circulating IGF-I levels are indicative of prostate cancer risk (reviewed by [136]).

It may be premature to assume that elevated circulating GH levels do not increase cancer risk at all, since these studies used relatively young men but prostate carcinoma is largely confined to elderly men. Moreover, numerous studies support the use of GH inhibitors such as somatostatin analogs, GHRH antagonists, or GHRH receptor antagonists in advanced cases of androgen-independent prostate cancer [141–145]. Larger prospective epidemiological studies are therefore required to fully evaluate the link between therapeutically or pathologically elevated GH titres and prostate cancer.

In contrast to the relatively benign effects of endocrine GH, recent studies suggest that prostatic GH plays a significant role in prostate tumorigenesis. Rodents with an intact GH axis expressing the C3(1)/T antigen (Tag) transgene invariably develop prostatic carcinoma [146]. However, disruption of the GH or GHR gene in Tag rodents decreases both the incidence and the severity of prostate malignancies, suggesting that GH is a necessary factor for prostate tumorigenesis [146, 147]. Similarly, human prostate cancer cell xenografts do not proliferate well when transplanted into GH-deficient lit/lit mice [148]. Surprisingly, Nakonechnaya et al. [149] observed that endocrine GH stimulated proliferation, but autocrine GH actually inhibited prostatic cell proliferation. The relevance of this finding to prostate tumorigenesis remains to be established.

GHRs and GHR mRNA are abundantly present in normal prostate tissue [110, 134] and prostatic carcinoma cell lines [134, 150–152]. At least in LNCaP cells, these receptors activate a signaling pathway implicated in the progression of prostatic tumors involving JAK2, AKT/PKB, and p42/p44 MAPK [152]. However, the role of this signaling pathway remains unclear, since GH-induced proliferation was observed by Untergasser et al. [150] but not by Weiss-Messer et al. [152].

GHR upregulation may be part of the tumorigenic process, since GHR expression is higher in cancerous prostate cell lines than in normal prostatic cell lines [134, 149], and GHR immunoreactivity in the prostatic epithelium of TAg rodents increases in parallel with tumor development [146]. Patterns of GHR autoregulation may partially account for differences between prostatic cell lines and, by extension, prostate tumours. For instance, prostate cell lines express a GHR isoform (GHRtr) lacking exon 9, which has a lower affinity for GH and generates more GHBPs than the full-length receptor [152]. The impact of GH on the relative expression of the normal and GHRtr isoforms varies between cell lines [153].

The GH gene is coexpressed with the GHR gene in both normal and cancerous prostate cell lines [151] and in human prostate tissue [154] and is upregulated in many prostate cancers (Oncomine). Chopin et al. [151] detected numerous GH transcripts, including those encoding the 20 kDa and 22 kDa variants of both the pituitary (GH-N) and placental (GH-V) GH proteins. Like the GHR, GH expression is higher in cancerous than normal prostate cell lines [149], and GH immunoreactivity increases in parallel with IL-6 expression and tumor progression [155].

The roles of hepatic and prostatic IGF-I as mediators of GH action remain unclear. Numerous studies support a role for circulating IGF-I in prostate growth (reviewed by [156]) and tumorigenesis (reviewed by [157]). Moreover, Ruan et al. [131] did not observe any effect of GH on prostate growth in IGF-I-null mice, and GH stimulates IGF-I gene expression in some, but not all, prostate carcinoma cell lines [158]. As a result of these and other studies, Bidosee et al. [158] concluded that GH potentiates estradiol- and IGF-I stimulated cell proliferation by stimulating IGF-I receptor and estradiol receptor synthesis, but it is ineffective alone. However, other investigations suggest that GH alters prostate function independently of IGF-I. For instance, serum and prostate IGF-I and prostate IGF-1 receptors do not increase during prostate tumor progression in TAg rats, and prostate IGF-I production actually decreases [146]. Moreover, the effects of GH and IGF-I on prostate enzyme production in rats are overlapping but distinct [133, 134].

Normal prostate growth and function require adequate concentrations of androgens, but numerous lines of evidence suggest that prostatic actions of GH are androgen-independent. For instance, the protective effect of GHR or GH knockdown on tumorigenesis in TAg mice is independent of changes in serum testosterone or prostate androgen receptors [146], and GH activates signal transduction pathways in prostate tumor cell lines but only transiently upregulates androgen receptor levels [152]. Moreover, unlike the association between PR and GH expression in mammary cell lines, a consistent relationship between GH and androgen receptor expression in prostate cancer lines has not been observed [6]. Moreover, androgens may actually suppress GH-induced tumorigenesis by upregulating the expression of SOCS2, an inhibitor of GH signaling [159]. The loss of SOCS2 signalling may contribute to prostate tumorigenesis [159].

#### 6.2.2. Penile Growth and Erectile Function

GH may be required for penile growth, since GH deficiency and GH resistance are frequently associated with micropenis [160]. Accordingly, GH therapy improves penile growth in GH-deficient boys [161, 162]. IGF-I may mediate this effect, since IGF-I administration to GH-resistant boys augments penile size, and this effect ceases when IGF-I therapy is withdrawn [60]. Similarly, Dykstra et al. [163] observed a stimulatory effect of IGF-I (but not GH) on the proliferation of cultured foreskin fibroblasts, independent of any changes in androgen receptors or 5-alpha reductase activity. However, a more recent study by Lee et al. [164] observed a stimulatory effect of GH on foreskin fibroblast proliferation that was at least partially mediated by local IGF-I.

In contrast, erectile function may be modulated by autocrine/paracrine GH. Erection requires modulation of blood flow and relaxation of penile smooth muscle. GH may facilitate both venous constriction and smooth muscle relaxation. The GH concentration in systemic and cavernous blood increases during penile tumescence in healthy men or men with psychogenic erectile dysfunction but does not increase in sexually aroused patients with organogenic erectile dysfunction [165, 166]. An earlier study, conversely, did not observe any changes in systemic GH concentrations during sexual arousal and orgasm [167].

GH improves the erection frequency and maximal intracavernous pressure in aged rats by stimulating nNOS expression in intracavernosal nerves [168, 169]. GH also improves the regeneration of nNOS-containing nerves following cavernous nerve neurotomy, accelerating the resumption of erectile function [170, 171]. This regenerative effect may involve local IGF-I and TGF-beta2, both of which were increased following GH stimulation [172]. NOS may mediate GH effects in humans, since GH, NO, and cGMP are tightly correlated in systemic and cavernous blood of individuals with erectile dysfunction [165] and GH induces both relaxation and cGMP production in human cavernous strips [173]. However, a later study showed that GH enhances cGMP signaling in human corpora cavernosa (isolated from transsexual patients receiving hormonal therapy) independently of NO [174].

Erectile effects of GH may be biphasic, since the pathophysiological GH concentrations in acromegalics are associated with erectile dysfunction [175] and GH at doses present in acromegalics stimulates contraction of dog corpus callosum strips [176]. The biphasic effects of GH on erectile function may partially reflect altered libido, since libido is impaired in acromegalics [175] and boars transgenic for the GH gene [177–179] as well as in GH-deficient males [180] and GHR-knockout mice [50].

## 7. Female Fertility

GH is a necessary factor for optimal female fertility, as evidenced by the decreased (but not absent) fertility in GHD women [181] and the ability of GH replacement to enable successful unassisted pregnancies in previously infertile GHD women [182]. Various fertility parameters are similarly reduced in GH-resistant GHR knockout mice (reviewed by [43]). In agricultural settings, a single GH injection (which elevates GH titers for 7 days [183]) at the time of either ovulation induction or insemination improves the pregnancy rate in cattle [184, 185]. However, as in males, high GH levels can inhibit fertility and promote neoplasms. These beneficial and detrimental effects of GH reflect the production and action of GH in the ovary and uterus.

### 7.1. The Ovary

The possibility that GH might enhance fertility by acting at the ovary (or upon ovarian components* in vitro*) has attracted significant attention in the last decade, with the aim of optimizing the successful outcomes from* in vitro* and* in vivo* fertilization protocols in agricultural and clinical settings. In addition to the fertility-related processes discussed below, GH also contributes to overall ovarian health, since its administration* in vivo* diminishes injury-induced tissue damage by acting as an antioxidant [186]. To our knowledge, no direct link has been found between GH and ovarian cancer. While GHRH antagonists are useful adjuvants in ovarian cancer treatment [187], the investigators did not implicate the GH axis in this effect.

The two ovarian processes required for normal fertility—oogenesis and folliculogenesis—depend upon an intricate system of intracrine, juxtacrine (via gap junctions), autocrine, paracrine, and endocrine signals. Of particular interest are the signals exchanged between the oocyte and granulosa cells that control early, gonadotropin-independent maturation and modulate and/or mediate the effects of gonadotropins upon later stages of folliculogenesis and oogenesis [188, 189]. As discussed in this section, GH, both of pituitary and ovarian origin, may be a modulatory signal in this complex interplay. While the processes of steroidogenesis, folliculogenesis, and oocyte maturation are discussed independently, they are intrinsically linked, and all must be optimized in order to produce a viable embryo.

#### 7.1.1. Steroidogenesis

Timely changes in ovarian estradiol and progesterone secretion are essential for all aspects of reproductive function, including follicular and luteal development, ovulation, and blastocyst development. It should also be noted that larger follicles/corpora lutea produce more steroids; thus, increased steroid production may reflect follicular size as well as altered enzyme activity.

Although pituitary gonadotropins are the primary regulators of ovarian steroidogenesis, the preponderance of* in vitro* evidence suggests that GH also modulates progesterone and estradiol release (reviewed by [3, 39]). For example, GH stimulates progesterone and estradiol production from bovine granulosa cells [190, 191] and human luteinized granulosa cells [192, 193]. GH effects vary throughout the ovarian cycle, since GH stimulates basal progesterone production in porcine corpora lutea but not follicles [194] and enhances leptin-induced progesterone production in follicles [195]. Karamouti et al. [192] observed a biphasic interaction, with low doses of leptin enhancing GH-induced estradiol production from human luteinized granulosa cells but high doses inhibiting the GH effect.

Nakamura et al. [196] observed a role for GH in FSH-induced but not basal steroidogenesis in rat granulosa cells, potentially mediated by antagonization of the BMP signaling system. In the presence of FSH, GH promotes early reactions in the steroidogenic pathway (such as StAR synthesis) via increased local IGF-I, thereby enhancing progesterone synthesis. Conversely, GH inhibits FSH-induced aromatase activity and thus estradiol synthesis, by an IGF-I independent pathway, since IGF-I alone stimulates aromatase activity. Similar results have been observed in Leydig cells (see references in Section 6.1.2). The fact that other investigators noted GH-induced increases in estradiol production (see references above) could reflect species and methodological differences, the stimulatory effects of GH on the early pathway steps overwhelming the inhibitory effect on the last step, and/or a concomitant increase in IGF-I resulting from GH treatment overwhelming the direct GH inhibitory action. In contrast, GH stimulates StAR expression in macaque MII oocytes independently of IGF1 and FSH [197].

*In vivo* studies of GH-induced steroidogenesis have produced inconsistent results. While some studies demonstrate increases in plasma estrogen or progesterone concentration (e.g., cattle [198] and pigs [199]), many others conclude that GH has either an inhibitory or no effect on ovarian steroidogenesis (reviewed by [3, 39]). A recent clinical study, for instance, correlated urinary GH with progesterone and estradiol concentrations, but it only observed an* in vitro* effect of IGF-I, not GH [200]. GH actions documented* in vitro* may be masked by other factors modified by exogenous* in vivo* GH administration, such as ovarian GH production. Nevertheless, the partial progesterone deficiency in GHR-deficient cattle suggests that GH is physiologically relevant to ovarian steroidogenesis [201]. The GH sensitivity of ovarian steroidogenesis is likely temporally dependent, since cGH* in vivo* increased ovarian estradiol and progesterone content prior to sexual maturity in chickens but only progesterone content at the time of sexual maturity [202]. Curiously, an* in vivo* study by Singh and Lal [203] observed augmented ovarian steroidogenesis following GH injections in the morning, but not in the evening.

#### 7.1.2. Folliculogenesis

Two of the most important oocyte-secreted factors (OSFs) in folliculogenesis are members of the TGF-beta superfamily, growth differentiation factor 9 (GDF-9) and bone morphogenic factor 15 (BMP-15) [204]. These OSFs (among others) direct the formation of cumulus cells from granulosa cells via a paracrine pathway and subsequently maintain and control these critical cells [205]. Also critical to their function is inhibiting progesterone production, thereby inhibiting luteinization [204]. The follicular actions of GH discussed below may reflect interactions with the BMP system, since GH downregulates BMP receptors and upregulates inhibitors of BMR signaling (Smad 6/7) [196]. Conversely, BMP signaling inhibits the formation of GHRs, IGF-I, and IGF-1Rs [196].

*In Vivo Studies*.* In vivo* studies indicate that the proliferative and antiapoptotic effects of GH extend to the ovarian follicle. Generally, GH administration increases follicular size and/or number (reviewed by [3, 39, 206]) and increases ovarian weight [203]. GH appears necessary for optimal follicular maturation and survival, since GHR knockout mice have more primordial follicles and fewer primary, secondary, preantral, and antral follicles, as well as increased follicular atresia [207, 208]. The impaired folliculogenesis in these GH-resistant animals results in lower ovulation and implantation rates, fewer corpora lutea, and smaller litter sizes [207].

In GH-replete animals,* in vivo* GH improves the number of developing follicles in young, but not aged, mice [209], in buffalo [210] and in superovulated sheep [211]. In chickens,* in vivo* GH promotes proliferation and inhibits apoptosis in the ovarian stroma and small (white) follicles [202]. GH similarly increases follicle size in undernourished cows [212]. In the latter study, exogenous GH may be neutralizing the inhibitory effect of suboptimal body condition on reproduction that is communicated by somatotropic axis suppression. Even in normally fed cows, the absence of GH action resulting from GH resistance completely blocks the development of the dominant follicle [201]. GH expression in transgenic mice [45] and ewes [213] enhances follicular development, increasing ovary weight, the ovulation rate, and/or the size and health of the ovarian follicles. Despite these apparent improvements in reproductive health, the conception rate and fetal survival are reduced in GH transgenic sheep, perhaps because of poor maternal glucose control [213].

The interpretation of these* in vivo* studies is complicated by the concomitant increase in circulating IGF-I induced by exogenous GH administration, since IGF-I exerts well-documented ovarian effects. Indeed, administration of exogenous GH results in an artificial situation in which both GH and IGF-I are elevated, whereas physiological increases in IGF-I normally result in a decrease in pituitary GH production. The absence of ovarian GHR mRNA observed in some studies, particularly in bovines, has led some investigators to conclude that “GH acts via other metabolic hormones, such as insulin and IGF-I, to influence follicular development” [188]. However, as discussed shortly, the extensive* in vitro* effects of GH on the ovarian follicle and the presence of ovarian GHR mRNA in most species suggest otherwise.

*In Vitro Studies*. Optimizing the* in vitro* maturation (IVM) of primordial and immature follicles is of clinical interest, in order to preserve the fertility of prepubertal girls undergoing chemotherapy. Moreover, IVM avoids or greatly reduces the costs and unpleasant side effects of gonadotropin-induced ovarian hyperstimulation associated with conventional* in vitro* fertilization (using oocytes matured* in vivo*). It is also of considerable agricultural interest, since the pool of immature follicles is much larger than that of mature follicles. However, while IVM oocytes can be successfully fertilized, their ability to produce viable offspring is limited [205]. Recent genetic analyses reveal that the transcription of numerous genes differs between oocytes matured* in vivo* and* in vitro*, including those encoding proteins modulating the interactions between oocyte and cumulus cells [214].

GH supplementation of the IVM medium may be part of the answer, since GH promotes the survival, activation, and growth of preantral follicles originating from goats [215, 216], sheep [217], and mice [218]. In mice preantral follicles, at least, GH promotes proliferation of both thecal and granulosa cells in these immature follicles [219]. GH similarly improves the oocyte retrieval rate and fertilization rate in human oocytes subjected to IVM but does not significantly alter the overall pregnancy rate [220]. Of particular interest is the ability of GH to promote cumulus expansion [221], since this may be a rate-limiting step in IVM. These studies were also able to use the GH-treated follicles in IVF protocols, resulting in the production of at least morulae [217] and in some cases live embryos [216]. Indeed, Izadyar et al. [222] and Mtango et al. [223] observed that the addition of GH to the IVM maturation medium enhanced the eventual number of cleaved embryos and blastocysts, while other studies observed similar numbers but increased quality [224–226]. Curiously, Shirazi et al. [227] observed that GH and FSH coincubation during IVM actually decreased the blastocyst rate, whereas each hormone alone promoted blastocyst production.

The activation of primordial follicles and their development into late preantral follicles is not gonadotropin-dependent and appears to rely upon a host of locally acting growth factors [228]. This effect may be mediated by the local growth factor GDF-9, since GDF-9 effectively stimulates preantral follicle development alone but does not act synergistically with GH [215]. Activin and other ovarian growth factors may mediate the actions of GH, since folliculostatin (which inactivates activin) blocks the proliferative response to GH in murine preantral follicles [218]. Leptin is also a candidate, since GH upregulates the long leptin receptor in prepubertal pig ovaries [229]. GH may play an important role in the development of follicles to the gonadotropin-dependent stage.

#### 7.1.3. Oocyte Maturation

The oocyte must undergo maturational processes before it can be successfully fertilized [230]. Nuclear maturation encompasses numerous sequential events, including the breakdown of the germinal vesicle and the resumption of meiosis, the first meiotic division, and the appearance of second metaphase chromosomes. These processes can be evaluated cytologically by the appearance of the first polar body or metaphase II chromosomes.

*In vivo* studies suggest that GH may improve nuclear maturation and thus oocyte quality. The addition of GH to an FSH-induced superovulation regime increases the percentage of ovulated follicles containing MII oocytes [209]. A contradictory study suggests that the combination of FSH and GH may have a negative effect on nuclear maturation, since overall ovulation rates were increased but the number of MII oocytes retrieved was unchanged in sheep receiving both FSH and GH [211].

Of significant interest is the possibility that GH might improve oocyte quality when administered* in vitro*. Accordingly, GH accelerates nuclear maturation in cumulus-oocyte complexes (COCs) of many species (e.g., bovine [223], canine [231], ovine [227], and equine [232]). Early GH exposure* in vitro* (prior to both IVM and IVF) also increases the percentage of oocytes resuming meiosis, suggesting that GH might establish optimal conditions for nuclear maturation, perhaps by promoting follicular development [216]. In equine COCs, the effect of GH is reduced in the presence of cAMP inhibitors [233].

Previous reviews postulated that GH affected nuclear maturation indirectly, by modulating cumulus cell expansion, since Apa et al. [234] observed a GH response in cumulus-enclosed oocytes but not in denuded rat oocytes. The degree of cumulus expansion is a strong predictor of oocyte quality, because cumulus cells play a critical role in oocyte nourishment and protection. GH enhances cumulus cell expansion by stimulating proliferation and inhibiting apoptosis [235, 236]. Cumulus cells also secrete various inhibitory factors that maintain oocytes in meiotic arrest via the cAMP signaling system [237]. These inhibitory factors may pass from surrounding cumulus cells to the oocyte through gap junctions, and the loss of connexin-43 gap junctions may release the oocyte to resume meiosis. GH may control the timing of meiotic resumption, since it upregulates connexin-43 expression in bovine granulosa cells from early follicles [34] but downregulates connexin-43 expression in maturing bovine COCs [236]. This effect is not observed in equine oocytes [238].

Conversely, the ability of GH to promote nuclear maturation of denuded oocytes from mice [239] and humans [240] suggests that GH also acts directly at the oocyte. GHR mRNA is readily detectable in oocytes as well as cumulus cells from many species, including human [241], rat [196], cow [236], horse [238], pig [242], and tilapia [118]. In the rhesus monkey [221] and rat [110], GHRs are present both in the oocyte cell membrane and within the ooplasm. In mature human ovaries, GHR immunoreactivity was also detected in the oocyte nucleus [243]. GHR expression increases during development and decreases at maturity in piscine [118, 244], porcine [245], and bovine oocytes [246], although not in human [241] or rhesus monkey [211], suggesting that these GHRs are physiologically relevant in some species at least.

Curiously, while both low (10 ng/mL) and high (100 ng/mL) GH improve nuclear maturation, only the low dose enhances cumulus cell expansion [221]. This result may reflect the peculiarities of GH:GHR stoichiometry; the higher dose may cause inactive complexes to form on cumulus cell membranes containing equal numbers of GH and GHR molecules. However, fewer GH molecules penetrate the cumulus layer to bind with oocyte GHRs, thereby maintaining optimal stoichiometry.

Cytoplasmic maturation occurs alongside of nuclear maturation, involving dramatic changes in oocyte gene expression, protein synthesis, and organelle organization [247].* In vitro* oocytes frequently complete nuclear maturation without completing cytoplasmic maturation, resulting in decreased fertilization and poor quality embryos after fertilization. Cytoplasmic maturation is conventionally evaluated based on the distribution of cortical granules, which become evenly dispersed in healthy metaphase II oocytes. The incubation of* in vitro* bovine [222] and equine [248] oocytes with GH significantly increases cortical redistribution.

#### 7.1.4. Ovulation Rate

The previously mentioned actions of GH on the follicle and oocyte likely impact the number of oocytes released in polyovulatory species, since the ovulation rate and resulting litter size are reduced in GHR-KO mice [44] and increased in GH transgenic mice [45]. The ovulation rate is also increased in GH transgenic sheep, but this increase did not translate into more live births due to increased fetal loss [213]. The addition of GH to superovulation regimens increases the abundance of retrieved follicles in sheep and thus may increase the number of gonadotropin-responsive follicles [211]. Daily GH administration to gilts similarly increases the ovulation rate [249], but long-term (at least 8-9 days) exposure suppresses subsequent estrus [250]. Conversely, high-level transgenic expression of the GH gene in pigs completely abolishes ovulation [178]. Extended premating GH treatment (about 80 days) did not affect ovulation or fetal survival but resulted in smaller fetuses [250].

GH administered at the time of superovulation induction increases the number of transferable embryos in FSH-treated ewes and lambs born per donor ewe, by decreasing the number of unfertilized eggs and degenerate embryos [251]. Conversely, Montero-Pardo and colleges [252] administered GH prior to superovulation and observed increased numbers of larger blastocysts but the increase did not translate into a greater percentage of transferable embryos.

#### 7.1.5. Luteal Function

The maintenance of the corpus luteum is critical for pregnancy viability. This postovulatory structure is effectively a transitory endocrine organ that maintains early pregnancy by secreting progesterone. It regresses at the end of nonproductive ovarian cycles or later in pregnancy by apoptosis. GHR mRNA and/or immunoreactivity are readily detectable in luteal cells from humans, rats, pigs, and cattle, implicating GH in luteal function [110, 253–255].

As expected, GH is proliferative and antiapoptotic in regard to the corpus luteum (reviewed by [3, 39]). For instance, GH stimulates the proliferation of human luteinized granulosa cells [254] and suppresses caspase-3 activity in bovine corpora lutea [194]. The proliferative and antiapoptotic effects are nevertheless distinct, since GHR knockout mice have fewer corpora lutea but also fewer apoptotic antral follicles [44]. Curiously, while most* in vivo* studies suggest that GH stimulates luteal growth and steroidogenesis in cattle [256], long-term GH infusion decreases luteal size and progesterone production [257].

The proluteal effects of GH may partially reflect GH-induced progesterone secretion (discussed above [258, 259]), since progesterone is antiapoptotic. However, other mediators have also been proposed. In cows* in vivo*, the continued survival of the corpus luteum relies on the embryonic secretion of IFN-*τ*, which inhibits uterine secretion of PGF-2*α*, which maintains the CL and prevents embryonic loss [260]. GH increases IFN-*τ*, perhaps by increasing embryonic growth [261]. GH also stimulates prostaglandin F2alpha production in the early bovine corpus luteum [262]. This maintenance of high endogenous PGF2alpha production maintains the corpus luteum directly, by rendering it insensitive to the luteolytic effects of exogenous PGF2alpha and, indirectly, by stimulating progesterone production [262].

#### 7.1.6. *In Vitro* Fertilization Protocols

Over 20 years ago, Owen et al. [263] concluded that GH improves the ovarian response to conventional ovarian stimulation regimens in women with poor ovarian responses (POR). This conclusion was based on studies by Homburg et al. [264] and others demonstrating that GH sensitizes the ovary to the ovulation-inducing actions of gonadotropins. Despite numerous more recent studies, the addition of GH to the IVF treatment regimens of poor responders remains controversial and “off-label” [265]. One complicating issue is the definition of a poor responder. According to the Bologna Criteria, a POR must meet at least two of these three conditions: (1) one or more risk factors for POR (including maternal age over 40); (2) a previous result in which fewer than three oocytes result from a conventional controlled ovarian stimulation (COS) protocol; and (3) an abnormal ovarian reserve test [266]. In support of this definition, the number of oocytes produced in response to gonadotropin stimulation (i.e., the ovarian response) can be prognostic of fertility [267, 268]. A recent review from de Ziegler et al. [265], however, questions the inclusion of condition of an abnormal ovarian reserve test, since they conclude that the most important determinant of artificial reproductive technology (ART) success is oocyte quality rather than oocyte quantity. Indeed, they conclude that the number of oocytes retrieved following ovarian stimulation is not necessarily a good indication of ART success. Meta-analysis of previous studies reveals that most links between poor ovarian response and ART failure simply reflect increased maternal age (which affects both quantity and quality). Oocyte quantity is not a useful predictor of ART success in patient populations other than older women.

As reviewed by Kolibianakis et al. [269] and Homburg et al. [270], earlier studies analyzing GH and ART have also been plagued by underpowered statistical analysis, the pooling of patients with diverse risk factors such as age, and heterogeneous protocols of GH administration, ovarian stimulation, and luteal support. Several large-scale meta-analyses, however, have addressed these drawbacks and concluded that GH is a useful* in vivo* adjuvant for human protocols. For example, Kolibianakis et al. [269] reanalyzed the results from multiple smaller studies and concluded that GH improves ART success, as indicated by an increased clinical pregnancy rate. Duffy et al. [271] similarly analyzed 10 studies and observed a statistically significant increase in the rates of pregnancy and live births when GH was included in the IVF protocol. Indeed, a recent meta-analysis by Kyrou et al. [272] determined that, out of all of the recently proposed protocol alterations, only GH supplementation and earlier embryo transfer significantly increased the IVF success rate.

The original study by Owen et al. [263] concluded that GH promotes oocyte quantity. Moreover, a study by Kucuk et al. [273] using a more extensive GH treatment regimen in a patient population with a significant proportion of smokers observed an improved ovarian response as well as increased ART success. However, most studies conclude that the outcomes improved by GH are generally those reflecting increased oocyte quality, such as the fertilization rate [273], the number of embryos reaching the transfer stage [269], the pregnancy rate [274], and the rate of live births [271]. A similar result was observed in a study targeting poor responders over 40 years old, in which GH was found to improve ART success (clinical pregnancy, implantation rates, delivery rates, and live-birth rates) without altering oocyte retrieval numbers [275]. GH is particularly effective in GH-deficient women, many of whom require GH therapy to induce normal ovulation [276]. GH supplementation to an ART protocol in this discrete population improves both the fertilization rate and the quality of the resulting embryos, as indicated by improved blastomere uniformity and cleavage rate and decreased apoptosis [277].

GH would thus appear to increase the efficiency of ART in poor responders and, in the meta-analyses of Kolibianakis et al. [269] and Duffy et al. [271], is not associated with any adverse events except for slight edema. Potential side-effects observed with GH treatment in other populations (such as aging males and GH-deficient adults) include increased fluid retention, resulting in edema, headaches, and/or joint pain, neoplasms, cerebrovascular events, and altered glucose metabolism (reviewed by Kokshoorn et al. [278]). The short duration of the GH treatment in ART, however, would suggest that these adverse effects would be quite rare. One potential downside of adjuvant GH therapy is cost, since Kolibianakis et al. [269] conclude that the addition of GH effectively doubles the treatment cost of ART. The potential that GH could cause long-term issues in the offspring could also be considered, since epigenetic changes in the embryo resulting ART are the topic of considerable current research [279, 280]. Official sanction of GH use in this context may also await additional large-scale studies, since the diversity of the population of PORs has led multiple authors to call for studies specifically addressing each subgroup (e.g., older women, polycystic ovary syndrome, and endometriosis) [265, 271].

#### 7.1.7. Ovarian Growth Hormone Production

While Silva et al. [206] suggest that systemic GH modulates ovarian function, granulosa cells and oocytes are avascular and separated from the systemic circulation by the basal lamina [281]. The ovarian actions of GH must thus be mediated either via ovarian stromal tissue or by locally produced GH. Indeed, GH expression is higher in the ovary than in the anterior pituitary or endometrium in goats and higher in prolific goats than in nonprolific goats [282].

GH mRNA and immunoreactivity are readily detectable in ovarian stromal and follicular tissue from numerous species, including bovine [283], porcine [245], chicken [284], and human [285]. Ovarian GH production is greater in the inner, largely avascular follicular compartments, since GH mRNA is detectable in granulosa cells and oocytes but is absent from cumulus cells and is less abundant in or absent from thecal cells [283, 284].

GH gene expression is initiated very early in follicular development in humans, since GH mRNA and immunoreactivity were detected in the oocyte cytoplasm and occasionally the granulosa cells of fetal primordial follicles [243]. GH mRNA was also detected in immature follicles of chickens [284] and pigs [245], but not in preantral follicles of rats [286] or cows [283]. Izadyar et al. [283] proposed that the GH gene expression increases during follicular development, since they detected GH transcripts in mature (but not immature) bovine follicles. Conversely Zhu et al. [245] observed a quantitative decline in porcine oocyte GH gene expression as immature follicles reinitiated meiosis. In any case, these temporal and spatial patterns of follicular GH expression parallel those of GHR expression in chicken [284], porcine [245], and human [243] follicles, adding further support to the possibility of autocrine/paracrine ovarian GH actions.

Modina et al. [287] observed a higher GH concentration in developmentally compromised bovine oocytes, suggesting that oocyte-specific factors also regulate GH expression. The authors reason that this upregulation is the cell's attempt to improve developmental competence. However, in human studies, follicular GH levels were positively correlated with ART success [275], and follicles containing higher GH levels gave rise to the highest quality embryos [288], so the link between GH production and oocyte quality remains unclear.

Local factors regulating ovarian GH synthesis remain elusive. Initial efforts focused on the traditional GH secretagogues such as GHRH and SRIF. GHRH mRNA [289] and GHRH receptor immunoreactivity [120] are present in human and rat [290, 291], although the rat GHRH transcript is distinct from the well-characterized hypothalamic transcript [291]. GHRH receptors, conversely, are notably absent from avian [292], bovine [283], and porcine [245] follicles, suggesting that GHRH does not control ovarian GH in these species. Ghrelin may instead act as the primary ovarian GH secretagogue, since ghrelin increases GH secretion but not synthesis in cultured whole porcine follicles [293]. The temporal increase in oocyte GH expression in some studies discussed above may partially reflect a local positive feedback loop between GH and ghrelin, since GH reciprocally stimulates ghrelin synthesis and secretion [293], although it does not affect GHS-R1a mRNA or protein levels [294]. Both ghrelin and the putative ghrelin receptor (GHS-R1a) have also been detected in the hen ovary [295], although their link to ovarian GH secretion has not yet been established.

#### 7.1.8. Involvement of IGF-I and IGF-II in Ovarian Actions

In addition to the extensive documented* in vitro* effects of GH, the finding of follicular GHR mRNA in many species further supports follicular GH actions independent of hepatic IGF-I [3, 39, 111]. GHR mRNA and/or binding activity is consistently higher in granulosa cells than in thecal cells, regardless of species (e.g., chicken [284], human [296], rat [196, 297], porcine [242], and bovine [246, 255]). The temporal pattern of GHR expression, conversely, is species-dependent, peaking during early folliculogenesis in pigs [242] and fish but not in humans [296] or rats [297]. In bovines, GHR mRNA expression is highest in estrogen-active dominant follicles, suggesting that GHR upregulation may be a turning point to enter the ovulatory phase [298]. This upregulation is independent of the concomitant increase in estrogen production but may be induced by FSH. GHR mRNA is present in preantral caprine follicles at levels equivalent to that of LHRs but significantly lower than FSHRs [216].

The involvement of local IGF-I in GH-mediated ovarian actions remains controversial and is likely species-specific. In support of its involvement, GH stimulates IGF-I expression in porcine [299] and rat [196] granulosa cells, via activation of the JAK/STAT pathway [196]. Also, IGF-1 antibodies (and cAMP blockers) block GH-induced oocyte maturation in rat follicles [300], and IGF-I administration improves follicular maturation in GHR knockout mice [207] and the maturation of mouse oocytes nonsynergistically with GH [239]. However, other lines of evidence suggest that GH and IGF-I act independently, at least in part. Follicular IGF-I levels are normal in GHR-knockout mice despite delayed follicular maturation [207, 208], likely reflecting the ability of other hormones (such as hCG and estradiol) to activate IGF-I production [301]. Also, IGF-1 antibodies cannot block the stimulatory effect of GH on cumulus cell expansion and oocyte maturation in bovine follicles [302], and, unlike GH, IGF-I cannot promote the maturation of primordial rat [228] or murine [218] follicles or the nuclear maturation of bovine COCs [236]. Arunakumari et al. [217] observed synergistic (and thus at least partially independent) effects of IGF-I and GH on the development of ovine preantral follicles. The coordinated upregulation of IGF2 and StAR in GH-incubated macaque follicles [197] and the ability of GH to stimulate IGF2 production in cultured human granulosa cells [303] suggest that IGF2, instead of or in addition to IGF1, may be an important follicular mediator of GH action, at least in primates.

### 7.2. GH and the Preimplantation Embryo

Despite major improvements in* in vitro* maturation and fertilization protocols, the success rate remains at about 33%, and* in vitro*-produced blastocysts show reduced quality compared to those fertilized and grown* in vivo* [304]. Embryos expressing an overactive GH gene polymorphism have an advantage* in vitro* but not* in vivo*, suggesting that GH helps overcome the effects of a suboptimal* in vitro* culture environment [305]. Embryonic quality is most vividly demonstrated by its survival after transfer, but earlier measures are highly useful for determining which embryo has the best chance of survival. Measures associated with posttransfer viability include the timing of the first cleavage (embryos cleaving earlier generally fare better), the cleavage rate, blastocyst diameter, and apoptosis in individual cells (blastomeres) [224].

GHRs are expressed in the 2-cell embryo, and GH in the blastocyst [306]. Incubation of 2-cell murine or bovine embryos in the presence of GH significantly increases the proportion of embryos successfully developing into blastocysts [225, 307–309]. In addition, GH-cultured blastocysts contain more blastomeres [308] and are of larger diameter [157]. The effects of GH are biphasic, with high concentrations inhibiting embryonic development [309], and are not observed in porcine embryos despite the presence of GH receptors [310]. GH mRNA is expressed consistently between the 2-cell and blastocyst stages [311]; thus, local GH may be acting in an autocrine or paracrine manner. Paracrine GH has been implicated in the enhanced growth of 2-day embryos cultured at higher density, since GH antibodies retard embryonic growth [309]. Of the two major cell populations in the blastocyst, GH appears to directly target the trophoblasts rather than the inner cell mass [309], despite the fact that GHRs are present on both cell types [307]. The trophoblast layer is critical for the formation of the blastocyst cavity as well as implantation (discussed shortly). IGF-I, conversely, appears to target the inner cell mass, and the lack of interference of antiserum against IGF-I or the IGF-1 receptor in GH actions suggests that IGF-I and GH act independently [225, 309]. Kölle et al. [312], conversely, concluded that GH reduced apoptosis of both trophoblasts and inner cell mass cells by reducing the expression of the antiapoptotic factor bax and, furthermore, that this effect was IGF-I dependent.

Equally important are the embryonic and maternal modifications necessary for successful embryonic implantation and placental formation, and 2-cell embryos incubated to the blastocyst stage in the presence of GH show higher levels of* in vivo* implantation [308]. The early blastocyst is surrounded by the zona pellucida, a protective matrix of glycoproteins and carbohydrates. The blastocyst must escape, or “hatch,” from this matrix before it can implant into the maternal endometrium [313]. GH improves the hatching rate in bovine [226] and murine [314] blastocyst populations. Increased production of matrix metalloproteinases (MMP) may potentially be involved, since these proteins play a role in implantation and are produced in response to GH (at least in liver) [315]. GH may also facilitate implantation by selectively stimulating trophoblast cell proliferation, since these epithelial-derived cells participate in blastocyst cavity formation and invasion of the maternal endometrium [309]. Furthermore, placental GH (and, to a lesser extent, pituitary GH) stimulates the invasive activity of these trophoblasts, and GHR expression is correlated with the degree of invasiveness [316]. An autocrine/paracrine interaction is possible, since invasive extravillous cytotrophoblast cells express GH-V mRNA and secrete placental GH [316].

Optimization of the maternal environment may be initiated very early in gestation, since a single, sustained-release injection of GH in sheep at breeding results in a smaller but more efficient placenta and heavier birth-weights [317]. Both endometrial gland development and secretory capacity are stimulated [318]. GH increases the amniotic glucose concentration and endometrial protein synthesis in* in vivo* pig embryos, suggesting a beneficial effect on nutrient transfer to the embryo [319]. Finally, pregnancy maintenance requires significant uterine hypertrophy. GH stimulates uterine growth, as discussed further below.

GH also alters the metabolic profile of* in vitro*-derived embryos to more closely resemble that of their* in vivo*-produced counterparts [320, 321]. These metabolic alterations may be critical for improvements in embryo quality. For instance, GH stimulates glucose uptake and glycogen utilization in preimplantation embryos [306, 321] and the maturational effects of GH are not observed in glucose-free medium [322]. Kidson et al. [310] suggest that the lack of GH effect on porcine blastocyst development reflects the glucose independence of early porcine embryo development, compared with the glucose dependence observed in other species. GH treatment of preimplantation embryos similarly reduces lipid accumulations, which may improve cryotolerance, and increases the number of specialized embryonic mitochondria, the abundance of which is strongly correlated with embryo quality [321].

Placental GH is the major isoform produced during gestation and exerts many wide-ranging effects on both mother and fetus. This increasingly complex field has been comprehensively reviewed elsewhere [323–328].

### 7.3. The Uterus

As mentioned above, the uterus is a site of both GH production and GH action. Both GH and GHRs are expressed in pregnant and nonpregnant uteri and GHRs are differentially regulated during pregnancy and the menstrual cycle compared with their hepatic counterparts [3]. For instance, GHRs are expressed in glandular cells of the human endometrium and decidua (but not stromal cells) during the mid and late luteal phases but not during the proliferative or early luteal phases [329]. In cattle, endometrial expression of GHR transcripts peaks at estrus through day 5 and subsequently declines [330]. While uterine IGF-I is abundant throughout gestation, its relevance to GH effects remains questionable. GH injection at breeding increases uterine IGF-I birth [317] but GH infusion in early gestation (day 16–30) does not [318].

GH is a uterine growth promoter, since GHD women have smaller uteruses than GH-replete women when corrected for body surface area [331]. Indeed, a normal GH axis may be necessary for estrogen-induced uterine hypertrophy, since uterine GHR mRNA abundance is strongly correlated with estrogen-induced uterine growth [332]. GH may thus facilitate implantation by stimulating uterine growth, since GHR-KO mice have fewer uterine implantation sites [44]. However, GH administration to GHD women did not appear to increase endometrial thickness [277].

In dogs, the most important ligand for uterine GHRs may be of mammary, rather than pituitary or uterine, origin. As reviewed by others (e.g., [6]), the canine mammary gland produces large amounts of GH, and mammary GH gene expression (but not uterine GH gene expression) is associated with endometrial hyperplasia [333, 334].

The mitogenic effects of GH have been implicated in uterine and cervical cancers. Autocrine GH may be particularly mitogenic, since uterine GH expression is upregulated in endometriosis and endometrial adenocarcinoma [155] and the degree of autocrine GH expression in endometrial carcinomas is strongly associated with the degree of tumor aggressiveness (as manifested by uterine invasion and the presence of ovarian metastases) [335].* In vitro* studies in an endometrial carcinoma cell line have subsequently revealed that the oncogenic potential of autocrine GH reflects increased anchorage-independent proliferation, cell survival, and cell migration and invasion [336]. Subcellular GHR distribution may be an important determinant of tumor aggressiveness, since nuclear GHR expression is associated with high proliferative activity [337]. Selective upregulation of nuclear (but not cytoplasmic) GHRs may be a contributing factor in the enhanced aggressiveness of cervical cancers in young patients [338].

GHRH and its receptor are also expressed in normal and tumorous endometrial tissue [339], and GHRH antagonists are promising chemotherapeutic agents for endometrial carcinoma [340]. However, GHRH antagonists may interfere with direct antiapoptotic effects of GHRH rather than suppressing endometrial GH production [341].

## 8. Conclusion

Growth hormone is intimately involved in reproduction in both males and females. For instance, it is critically required for timing the onset of puberty and the induction of sexual maturation. It also regulates the growth and actions of secondary sexual tissues, activating the uterus in females and the prostate and seminal vesicles in males. In adults, GH modulates gonadotropin secretion and it exerts gonadotropin-dependent and gonadotropin-independent actions on gonadal function, including steroidogenesis and gametogenesis. It also promotes ovulation and corpus luteum function, as well as improving the development of the preimplantation embryo. Growth hormone also improves placentation and is progestational. It is additionally mammogenic and galactopoietic. These actions promote fertility in both males and females and partially reflect the endocrine roles of pituitary GH, but as reproductive tissues are not just sites of GH action but also sites of GH synthesis, they may reflect autocrine, paracrine, and intracrine actions of GH produced within the reproductive system.
